# Introduction of Multiple CeO_2_ Interlayers to Avoid Ni Agglomeration in Nanoscale Ni–YSZ Solid Oxide Cell Hydrogen Electrodes

**DOI:** 10.1021/acsami.5c08989

**Published:** 2025-07-16

**Authors:** Mustafa Ünsal Ünver, Sorour Semsari Parapari, Sašo Šturm, Aligul Buyukaksoy

**Affiliations:** † Department of Materials Science and Engineering, 52962 Gebze Technical University, 41400 Kocaeli, Turkey; ‡ Department for Nanostructured Materials, Jožef Stefan Institute, 1000 Ljubljana, Slovenia; § Jožef Stefan International Postgraduate School, 1000 Ljubljana, Slovenia; ∥ Department of Geology, Faculty of Natural Sciences and Engineering, University of Ljubljana, 1000 Ljubljana, Slovenia

**Keywords:** solid oxide cells, nanoscale electrodes, Ni–YSZ, CeO_2_ interlayers, Ni agglomeration

## Abstract

Nanostructuring of solid oxide cell (SOC) electrodes is necessary for enhancing the electrochemically active triple phase boundary length and thus lowering the operating temperature of these devices. However, nanoscale morphology in nickel–yttria-stabilized zirconia (Ni–YSZ) electrodes renders the structure extremely prone to Ni agglomeration and hence performance degradation upon long-term operation. To overcome this challenge, application of multiple CeO_2_ nanolayers into the nanoscale Ni–YSZ electrodes, all produced from direct deposition of liquid precursors, is proposed. This way, constraining the movement of Ni, while maintaining electronic and ionic transport within the electrodes, is aimed. Microscopy analyses reveal relatively stable microstructures in the case of electrodes with an optimized number of CeO_2_ interlayers after ca. 100 h exposure to dilute hydrogen flow at 650 °C. Meanwhile, severe Ni agglomeration is observed in reference nanoscale N–iYSZ electrodes. In accordance, the ca. 10^5^-fold increase in the electrode polarization resistance of bare Ni–YSZ under the same conditions is reduced to ca. 50% increase in the case of CeO_2_ interlayer containing electrodes. The results presented here provide an effective method to implement nanoscale Ni–YSZ electrodes in intermediate-temperature SOCs while retaining microstructural stability.

## Graphical abstract

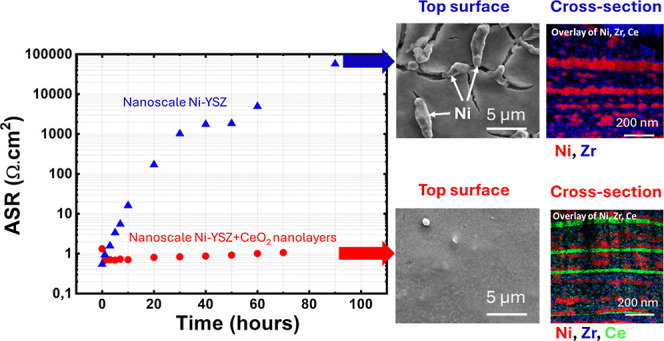

## 1 Introduction

Solid oxide cells are electrochemical devices that can operate at high efficiencies to produce electricity or hydrogen in fuel cells or electrolysis modes, respectively. Nickel–yttria-stabilized zirconia (Ni–YSZ) composites are the state-of-the-art fuel electrode materials for SOCs. Fabrication of conventional Ni–YSZ fuel electrodes is based on mixing electrocatalyst NiO and ionic conductor YSZ powders, followed by their cosintering at high temperatures (1300–1500 °C). NiO is reduced to metallic Ni under the reducing cell operating conditions.^1–4^

To achieve a high performance from Ni–YSZ electrodes, maximizing the length of the electrocatalyst-ionic conductor-gas triple phase boundaries (TPBs—where the hydrogen oxidation or hydrogen evolution reactions take place) is desired. Researchers have attempted to realize this goal through particle size reduction by using different fabrication methods. For example, Marinšek et al. synthesized nanocomposite NiO–YSZ powders by a combustion route,^5^ while Xi et al. used the coprecipitation technique to achieve small particle NiO and YSZ particle size.^6^ All of these synthesized fine powders required high temperature sintering to ensure strong mechanical contact at the NiO/YSZ interface within the electrode and at the electrode/electrolyte interface.

Another approach to Ni-based composite electrode fabrication was the infiltration of Ni-containing liquid precursors into presintered, porous ionic conductor scaffolds, followed by low-temperature (300–400 °C) drying.^7–13^ The fact that the infiltrated Ni electrocatalyst phase is not exposed to higher temperatures than those during operation contributes to the achievement of small particle sizes (Ni: 20–200 nm, YSZ: 200 nm–2 μm) and electrode polarization resistances as low as below 0.1 Ω·cm^2^ at 800 °C.^7,9^

For even smaller average particle sizes and thus further improved electrochemical performances, powder-free methods based on film deposition techniques have been explored. For example, Muecke et al. fabricated nickel–gadolinia-doped ceria (Ni–GDC) nanocomposite films on dense electrolytes by pulsed laser deposition (PLD) and spray pyrolysis of Ni-, Gd-, and Ce-containing liquid precursors.^14,15^ Average Ni and GDC particle sizes in the 16–53 nm range, which yielded polarization resistances of 0.34 Ω·cm^2^ at 600 °C, were obtained.^14^ Adopting a similar approach, polymeric precursors containing Ni, Zr, and Y cations were deposited onto YSZ electrolytes by spin-coating and low-temperature heat treatment at 400–800 °C, which induced the self-assembly of Ni and YSZ phases consisting of ca. 20 nm sized particles.^16,17^ Electrode polarization resistances as low as 0.64 Ω·cm^2^ at 550 °C were obtained.^16^

Regardless of the method used to fabricate them, Ni-based composite SOC electrodes have been associated with a serious Ni agglomeration problem, which occurred upon long-term operation, diminishing the TPB length and hence, the electrochemical performance.^15,18–22^ A surface energy-driven, solid-state (bulk or surface) diffusion-mediated Ostwald Ripening process^20,23^ as well as gas phase transport of Ni­(OH)_*x*_ compounds^23,24^ have been considered as possible mechanisms through which Ni agglomeration took place.

In Ni-based nanocomposites fabricated by film deposition methods, Ni agglomeration was reported to be extremely pronounced, even at ≤ 650 °C,^15,17,25^ and was accompanied by large Ni particle formation at their outer surface.^15,17,25^ For example, Bilbey et al. reported a polarization resistance increase from 1.19 to 163.9 Ω·cm^2^ in 100 h at 600 °C in nanoscale Ni–YSZ films.^17^ Muecke et al. proposed that for Ni agglomeration to take place, it should mechanically crush the constraining ionic conductor phase (gadolinia-doped ceria—GDC) network within.^15^ They also argued that, close to the electrode surface, there is less mechanical constraint and thus, growth of large Ni particles could occur.^15^ Application of a sintering process in the oxidized state prior to testing resulted in a mechanically stronger GDC^15^ or YSZ^17^ skeletons, which confirmed the mechanical constraining role of the ionic conductor phase in slowing down Ni agglomeration. Our recent study showed that the application of a CeO_2_ overlayer onto the Ni–YSZ thin film electrode surfaces prevented the large particle formation at the surface and enhanced microstructural and electrochemical performance stability.^17^

In their calculations, Qiu and Guzonas showed that under the application conditions of nanocomposite Ni–YSZ film electrodes (i.e., 9.72% H_2_–87.51% Ar–2.77% H_2_O, at ≤ 650 °C), metallic Ni should be thermodynamically stable and it would take a much higher amount of H_2_O for it to convert to Ni­(OH)_2_.^26^ Also, the fact that strengthening the ionic conductor network and the application of a constraining oxide overlayer stabilized the microstructure suggests that for nanoscale Ni-based composite electrodes at ≤ 650 °C and low (ca. 3%) humidities, vaporization of Ni is limited, and its agglomeration occurs via solid-state diffusion/migration. With this assumption in mind, here, we hypothesize that large Ni particle formation does not occur only by the Ni phase close to the surface with enough available space, but a general migration of Ni phase from the bulk of the film toward the electrode outer surface also takes place. To test this hypothesis and prevent Ni agglomeration within the film and at the outer free surface, for the first time in the literature, we propose to apply multiple CeO_2_ nanolayers into the Ni–YSZ electrode (see a schematic representation in Figure 1). Enabled by the layer-by-layer nature of the polymeric precursor deposition-based fabrication approach, we envision that the application of the CeO_2_ interlayers would inhibit Ni agglomeration due to the stable oxide form under operating conditions while not adversely affecting the electrochemical performance due to mixed ionic electronic conductor (MIEC) properties of the interlayer material.^27,28^ We believe that the outcomes of this study will allow the achievement of thermally stable nanostructured Ni-based composite electrodes that can be applied to intermediate-temperature SOCs.

**Figure 1:**
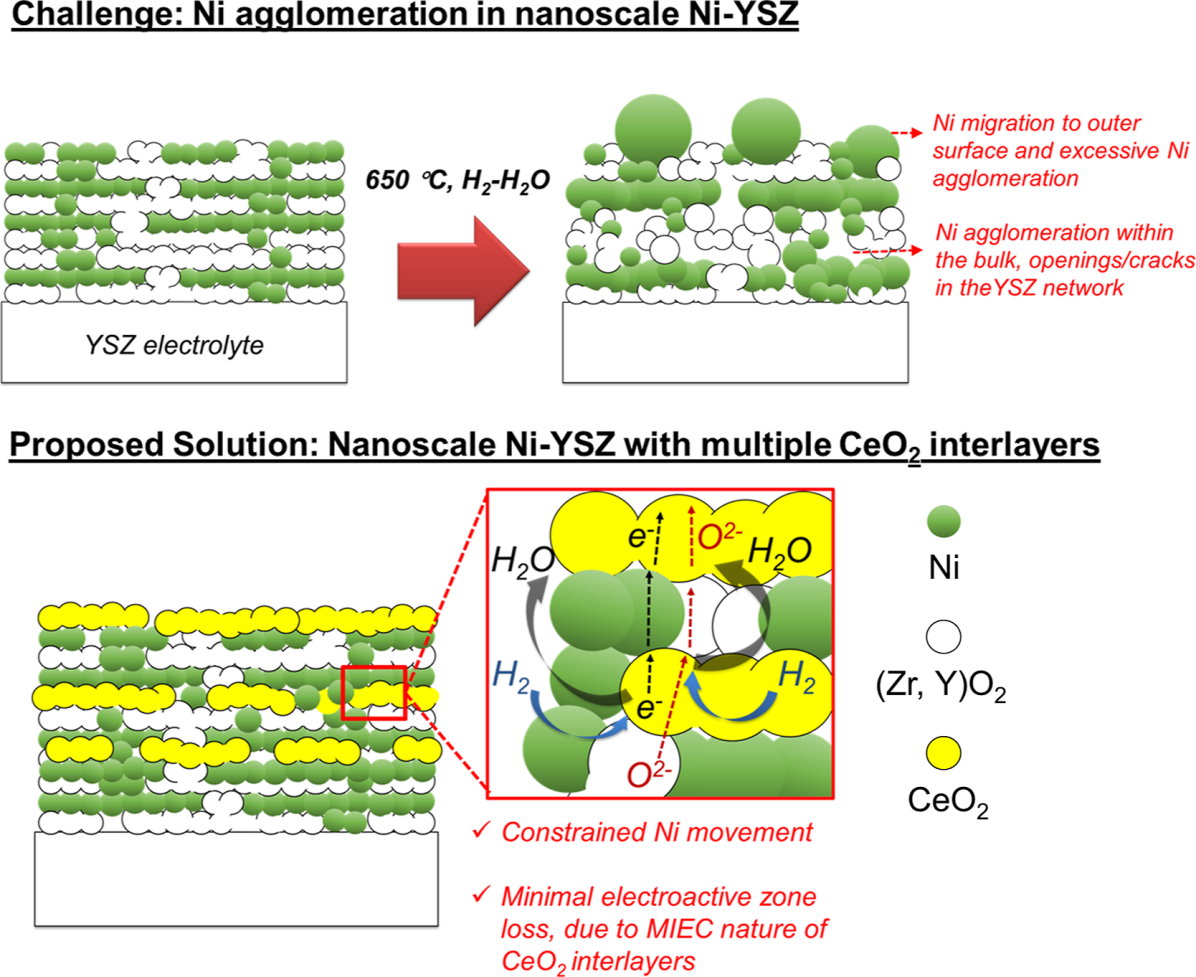
Schematic representation of the hypothesis stating that performance degradation mechanisms in fuel electrodes will be prevented by introducing multiple interlayers. Addition of multiple CeO_2_ interlayers should provide both a chemically stable mechanical constraint to Ni agglomeration while still allowing electronic and ionic transport due to the mixed ionic electronic conductivity of the selected interlayer material.

## 2 Materials and Methods

### 2.1 Sample Fabrication

To fabricate Ni–YSZ electrode films with nanoscale morphology, a powder-free approach involving the spin-on deposition and drying of a single liquid precursor containing Ni, Zr, and Y cations was adopted.^17^ To prepare the liquid Ni precursor, nickel­(II) nitrate hexahydrate (Ni­(NO_3_)·6H_3_O, Sigma-Aldrich) was dissolved in deionized water. Then, ethylene glycol (99% Alfa Aesar) was added to the solution in appropriate amounts to set the molar ratio of cations to ethylene glycol at 0.04. The resultant solution was then stirred on a hot plate at 80 °C until all of the added water evaporated. The YSZ precursor was prepared the same way. As Zr and Y sources, yttrium­(III) nitrate hexahydrate (Y­(NO_3_)­6H_2_O, Alfa Aesar, 99.9%) and zirconium­(IV) oxychloride octahydrate (ZrOCl_2_·8H_2_O, Sigma Aldrich, 99.5%) were used, respectively, at a Y:Zr molar ratio of 0.16:0.84. The total cation:ethylene glycol molar ratio was 0.02 in this case.

Ni and YSZ precursors were then mixed to obtain a single clear solution. The mixing ratio was set to ensure that upon deposition, drying, calcination, and testing, the Ni to YSZ volume ratio would be 60:40. Finally, to achieve a solution with a low surface tension, 2-butoxyethanol (Sigma Aldrich) was added in equal amounts to the total solution. The obtained single solution was spin-coated onto dense YSZ substrates, which were fabricated by die pressing YSZ (TOSOH-8Y) powder and sintering at 1400 °C, for 4 h. The spin-on deposition was carried out via an EZ4 Spin coater, Schwan Technology, using a spinning speed of 3000 rpm. After the deposition, a 10 min drying step was carried out at 350 °C. This deposition/drying cycle was repeated 30 times on both sides of the electrolyte to obtain symmetrical electrode/electrolyte/electrode symmetrical cells. This way, a reference sample with no CeO_2_ interlayers (REF-Ni–YSZ) was obtained.

To achieve nanoscale Ni–YSZ electrodes with multiple CeO_2_ interlayers, spin-on deposition of a liquid CeO_2_ precursor (prepared using the same route as other precursors but using cerium­(III) nitrate hexahydrate (Ce­(NO_3_)_3_·6H_2_O)), followed by a drying step, was introduced into the Ni–YSZ thin film electrode fabrication procedure. The number of CeO_2_ interlayers was varied while keeping the total thickness of the electrode comparable. For example, the sample encoded as ML-101 is achieved via applying 10 deposition/drying cycles of the Ni–YSZ precursor, followed by 1 CeO_2_ precursor deposition/drying cycle. This procedure was repeated three times, which led to an electrode with a total of 3 CeO_2_ interlayers (ML-101). The number of CeO_2_ interlayers was further increased by depositing the CeO_2_ precursor every 7 (ML-71), 5 (ML-51), and 3 Ni–YSZ depositions (ML-31). To observe the effect of the CeO_2_ interlayer thickness, one sample was fabricated by depositing 3 CeO_2_ layers after every 5 Ni–YSZ depositions (ML-53). Another sample was fabricated by switching the CeO_2_ interlayer composition from 100% CeO_2_ to 70 vol % CeO_2_–30 vol % Ni mixture. This was realized by mixing appropriate amounts of the Ni precursor into the CeO_2_ precursor. Three layers of CeO_2_–Ni depositions were carried out after every 5 Ni–YSZ depositions (ML-53-Ni). A summary of the sample properties is provided in Table 1 and schematically in Figure S1.

**Table 1:** A Summary of Sample Preparation Parameters^a^

sample name	REF-Ni–YSZ	ML-101	ML-71	ML-51	ML-31	ML-53	ML-53-Ni
number of Ni–YSZ depositions prior to each CeO_2_ interlayer deposition	NA	10	7	5	3	5	5
number of depositions to construct one CeO_2_ interlayer	NA	1	1	1	1	3	3
composition of the CeO_2_ interlayer (CeO2:Ni volume ratio)	NA	100:0	100:0	100:0	100:0	100:0	70:30
total number of CeO_2_ interlayers	0	3	4	5	8	4 × 3	4 × 3
total number of Ni–YSZ or CeO_2_ depositions	30	33	32	30	32	32	32
estimated total Ni–YSZ layer thickness (nm)	900	900	840	750	720	600	600
estimated total CeO_2_ layer thickness (nm)	0	54	72	90	144	216	216

a Total Ni–YSZ and CeO_2_ interlayer thicknesses in all samples were determined by measuring the thicknesses of these layers (i.e., thicknesses of the CeO_2_ layers fabricated by one deposition and the thicknesses of Ni–YSZ layers fabricated by five depositions) on the TEM images and multiplying these values by the total number of CeO_2_ and Ni–YSZ depositions.

Before the electrochemical testing, all symmetric half-cells that were produced with an electrode/electrolyte/electrode configuration were heat treated for 4 h at 800 °C after deposition.

### 2.2 Materials Characterization

To investigate the microstructural evolutions of reference Ni–YSZ and Ni–YSZ with CeO_2_ multilayers upon long-term exposure to 650 °C under fuel gas flow (9.72% H_2_–87.51% Ar–2.77% H_2_O), a scanning electron microscope (SEM; Philips XL 30 SFEG) was used to observe and analyze the microstructure at various magnifications.

A more detailed microstructural, structural, and chemical characterization of the Ni–YSZ samples with and without CeO_2_ multilayers was carried out by (scanning) transmission electron microscopy ((S)­TEM). For this analysis, first, an electron-transparent lamella was prepared by the focused-ion beam (FIB) technique. A FIB-SEM Helios NanoLab 600i Dual Beam System (Thermo Fisher Scientific, Waltham, U.S.A., previously FEI) was used for this purpose. An area at the top surface of each sample was selected to extract a lamella containing the multilayers. The selected area was covered with a thin layer of Pt (∼3 μm) to protect the sample from damages induced by the Ga^+^ ion beam. In the first steps, high ion voltages and currents (30 kV and 21 to 0.77 nA) were used to detach the lamellae from the bulk sample. The lamella was then attached to a copper TEM grid, where it was thinned and polished to a final thickness of about 50 nm using low ion voltages and currents (16 to 1 kV and 0.77 nA to 21 pA).

The prepared FIB lamella was then characterized using a probe Cs-corrected JEM-ARM 200CF TEM (JEOL, Tokyo, Japan) equipped with a JEOL Centurio 100 mm^2^ energy-dispersive X-ray spectroscopy (EDS) detector, operated at 200 kV. The TEM images and the selected area electron diffraction (SAED) patterns were collected using an UltraScan 1000 CCD array detector (GATAN, Pleasanton, USA). EDS elemental maps were produced in scanning TEM mode (STEM–EDS) to evaluate the elemental distribution with a high spatial resolution. For structural analysis of the electron diffraction patterns, the structures were modeled using the Single Crystal software (CrystalMaker Software Limited, Oxfordshire, UK) and with the crystallographic information extracted from the Inorganic Crystal Structure Database (ICSD).

ImageJ software^29^ was used to analyze the SEM images and some of the TEM images to determine the microstructural features such as percent coverage of the surface Ni particles on the electrode surface and the porosity of the electrodes.

### 2.3 Electrochemical Characterization

Electrochemical impedance spectroscopy (EIS) (Gamry Potentiostat, Reference 3000, Warminster, USA) was used to test the electrochemical performance of the symmetric half-cells. EIS measurements were performed under open-circuit conditions in the frequency range of 5 × 10^–2^ to 1 × 10^5^ Hz using an excitation voltage of ±10 mV. Electrochemical performance tests were carried out under humidified 10% H_2_–90% Ar gas flow at 650 °C for 100 h. The bubbling water temperature was measured as 23 °C, which corresponds to 2.77% humidity, yielding a gas mixture of 9.72% H_2_–87.51% Ar–2.77% H_2_O. An in-house Ni paste, prepared by mixing commercial nickel oxide powder (99%; Alpha Aesar), α-terpineol (>96%), and 2-butoxyethanol (Sigma Aldrich), was used as a current collector. Nickel wire (Alpha Aesar, 0.25 mm diameter, 99.98%) was attached to the half-cells by the application of small amounts of Ceramabond (Aremco 552) at the edges of the sample—a location far from the electrodes. This way, contact between the electrodes—current collecting layers—and the lead wires was established.

## 3 Results

To determine the effect of multiple CeO_2_ interlayers on the microstructural stability of the nanoscale Ni–YSZ electrodes, scanning electron microscopy (SEM) analyses were performed on the top surfaces of as-deposited and calcined (800 °C, air, 4 h), as-reduced (650 °C, 9.72% H_2_–87.51% Ar–2.77% H_2_O gas, 2 h), and long-term annealed (latter conditions, 100 h) samples with varying numbers of CeO_2_ interlayers (Figure 2). NiO-YSZ films both with and without CeO_2_ interlayers homogeneously covered the YSZ substrate (Figure 2a–e). In the as-reduced form, the REF-Ni–YSZ surface had several cracks (Figure 2f). Introduction of CeO_2_ interlayers in increasing numbers reduced the number of cracks in the as-reduced Ni–YSZ electrodes (Figure 2g–i), yielding a crack-free surface in the ML-51 case (Figure 2i). However, a further increase in the number of CeO_2_ interlayers, again, induced crack formation (ML-31, Figure 2j).

**Figure 2:**
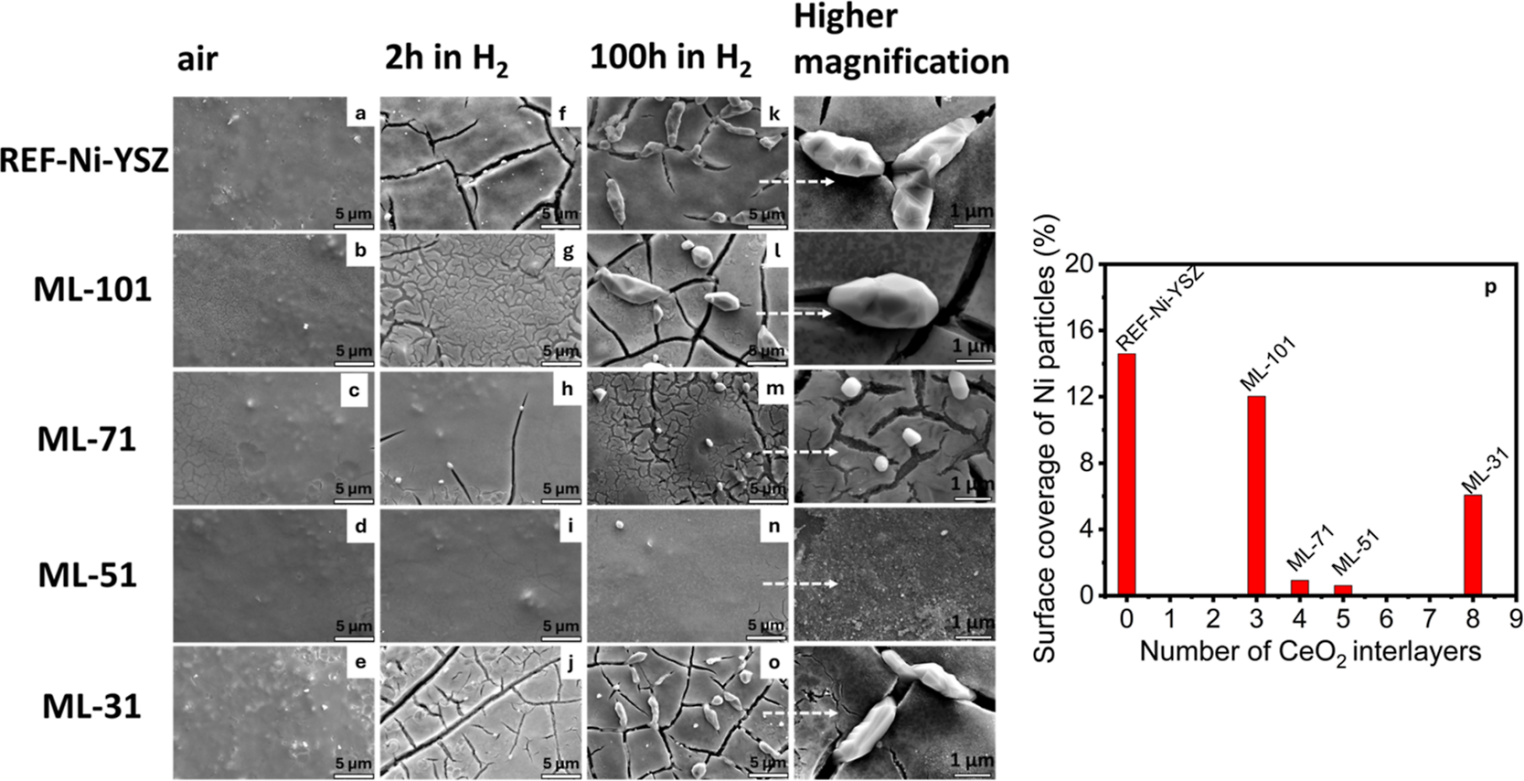
Top surface SEM images of samples in their as-calcined (a–e) and as-reduced states (f–j) and those collected after 100 h of exposure to 9.72% H_2_–87.51% Ar–2.77% H_2_O at 650 °C (k–o). (p) shows the percent area coverage of the surface Ni particles for each sample, determined from ImageJ analysis.

Upon 100 h exposure to 9.72% H_2_–87.51% Ar–2.77% H_2_O gas, at 650 °C for 100 h, large (few microns in size) Ni particles oozing out from the surface cracks formed (Figure 2k). With the increasing number of CeO_2_ interlayers, the number and size of surface Ni particles decreased (Figure 2l–n), finally yielding a Ni particle-free surface in the case of ML-51 (Figure 2n), after long-term annealing. Further increasing the number of CeO_2_ interlayers, again, induced Ni particle formation at the surface, all of which were colocated with a crack (Figure 2o). Figure 2p shows the percentage of the surface area of the electrodes covered by the surface Ni particles, determined through ImageJ processing.

In addition to the Ni particle formation at the top surfaces of the electrodes, the impact of the multiple CeO_2_ interlayers within the Ni–YSZ films was also investigated by examining cross-sectional FIB lamellae in the multilayer regions. The microstructural and structural evolutions were analyzed by acquiring high-angle annular dark-field STEM (HAADF-STEM) images and SAED patterns by TEM. The compositional evolution of Ni, YSZ, and CeO_2_ particles at the nanoscale was investigated by collecting STEM–EDS elemental maps.

Two samples were selected for TEM analysis. First, the sample with the most drastic microstructural changes at its surface (REF-Ni–YSZ), and second, the sample with the most stable surface morphology (ML-51). Figure 3 compares the HAADF-STEM image, SAED pattern, and EDS elemental maps of Ni, Zr, and Ce in the multilayer area for these two samples. The presence of a layered microstructure consisting of horizontally aligned layers can be seen for both samples on the HAADF-STEM images. The SAED patterns were obtained from the marked areas (dashed circles) on the HAADF-STEM images. The simulated diffraction patterns of Ni (ICSD no. 53808 ^30^), YSZ (ICSD no. 60393 ^31^), and CeO_2_ (ICSD no. 155604 ^32^) are superimposed on the experimental SAED patterns, respectively, as red, blue, and green quarter circles. The SAED ring patterns of both samples show that both multilayered regions were composed of polycrystalline grains. Sample REF-Ni–YSZ (Figure 3a) contains Ni and YSZ layers, while sample ML-51 (Figure 3b) additionally contained CeO_2_ layers, as shown by the indexed planes on the patterns. The elemental STEM–EDS maps acquired in the corresponding STEM images are also shown in Figure 3a and 3b for sample REF-Ni–YSZ and sample ML-51, respectively. Ni, Zr, and Ce distribution maps are shown by red, blue, and green colors, respectively. Sample REF-Ni–YSZ consisted of alternating Ni and YSZ layers, while ML-51 also had ca. 20 nm-thick CeO_2_ layers deposited separately (i.e., not from the same, single precursor that contained Ni, Zr, and Y cations). We note that REF-Ni–YSZ did not contain the CeO_2_ phase, and the Ce map is only shown for comparison reasons. The EDS maps indicate that phase separation of nickel (Ni)- and yttria-stabilized zirconia (YSZ) occurred after the long-term heat treatment of both REF-Ni–YSZ and ML-51 samples. The REF-Ni–YSZ sample exhibited thicker Ni layers, measuring up to approximately 50 nm in thickness, compared to the ML-51 sample, which had Ni layers around 20 nm thick. Additionally, after 100 h of annealing, the REF-Ni–YSZ sample displayed smaller isolated Ni particles.

**Figure 3:**
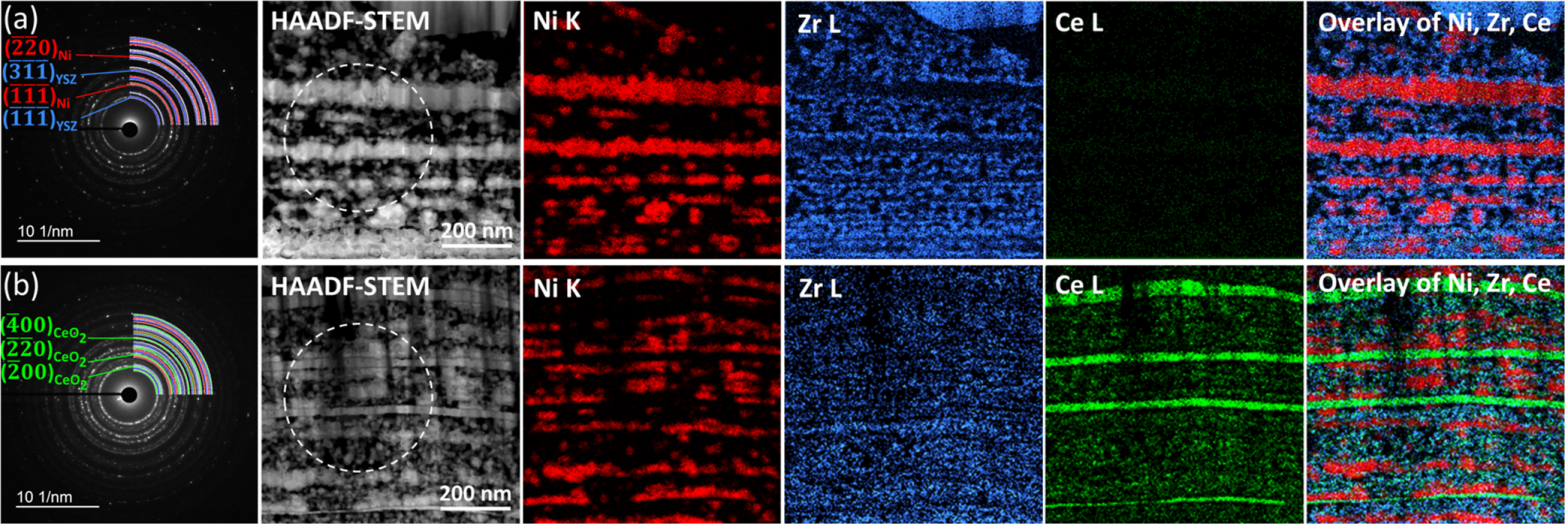
HAADF-STEM images, SAED patterns, and STEM–EDS elemental maps obtained at the cross-sectional view at the multilayered region of (a) REF-Ni–YSZ and (b) ML-51 samples (exposed to 9.72% H_2_–87.51% Ar–2.77% H_2_O at 650 °C). The SAED patterns were collected at the marked dashed circles on the HAADF-STEM images. The simulated patterns of Ni, YSZ, and CeO_2_ are superimposed on the experimental SAED patterns with red, blue, and green quarter circles, respectively.

TEM images collected from REF-Ni–YSZ and ML-51 samples after long-term annealing under operating conditions were analyzed by ImageJ (Figure S2). Their porosities were determined, respectively, as 34.4 and 34.0%. Considering their molar volumes, reduction of NiO to Ni should generate a ca. 40% volume reduction.^33^ Also, taking into account that Ni is about 60 vol % of the whole electrode and that additional porosity may have occurred due to incomplete sintering upon heat treatment at 800 °C, these values can be regarded sensible.

To evaluate the impact of the application of CeO_2_ interlayers on the electrochemical performance of Ni–YSZ nanocomposite electrodes, electrochemical impedance spectroscopy (EIS) measurements were conducted on symmetrical half-cells at 650 °C, under the flow of 9.72% H_2_–87.51% Ar–2.77% H_2_O gas and at an open-circuit voltage (Figure 4). In general, Nyquist plots of all samples consisted of two semicircles, each semicircle corresponding to one electrochemical process with a discrete frequency (Figure 4a). The high frequency intercept was ascribed to ohmic electrolyte and current collection resistances (Figure 4a). Equivalent circuit fittings were performed on the EIS data gathered from the samples after 3 h of exposure to 650 °C to ensure steady-state conditions (data presented in Figure 4a). Since the EIS data collected from all samples consisted of two semicircles, it was appropriate to use equivalent circuits that consisted of three serial resistors, two of them connected in parallel to a constant-phase element each (ASR_ohmic_–ASR1/Q1-ASR2/Q2) for fittings. Fit lines are shown in Figure 4a, while the area specific resistance and capacitance (ASR and ASC, respectively) values extracted from the fitting procedures are listed in Table 2. The total distance between high and low frequency intercepts, i.e., the sum of ASR1 and ASR2, is the total polarization resistance of the two electrodes (ASR_total_). To determine the polarization resistance per electrode (ASR_electrode_), the ASR_total_ was divided by two. REF-Ni–YSZ had an ASR_ohmic_ value of 22.2 Ω·cm^2^, which decreased when multiple CeO_2_ interlayers were introduced (Table 2). Only in the case of ML-31, the ASR_ohmic_ value went back up to 25.7 Ω·cm^2^. ASR1 was larger than ASR2 in all cases and exhibited a trend similar to that of ASR_ohmic_ (Table 2).

**Figure 4:**
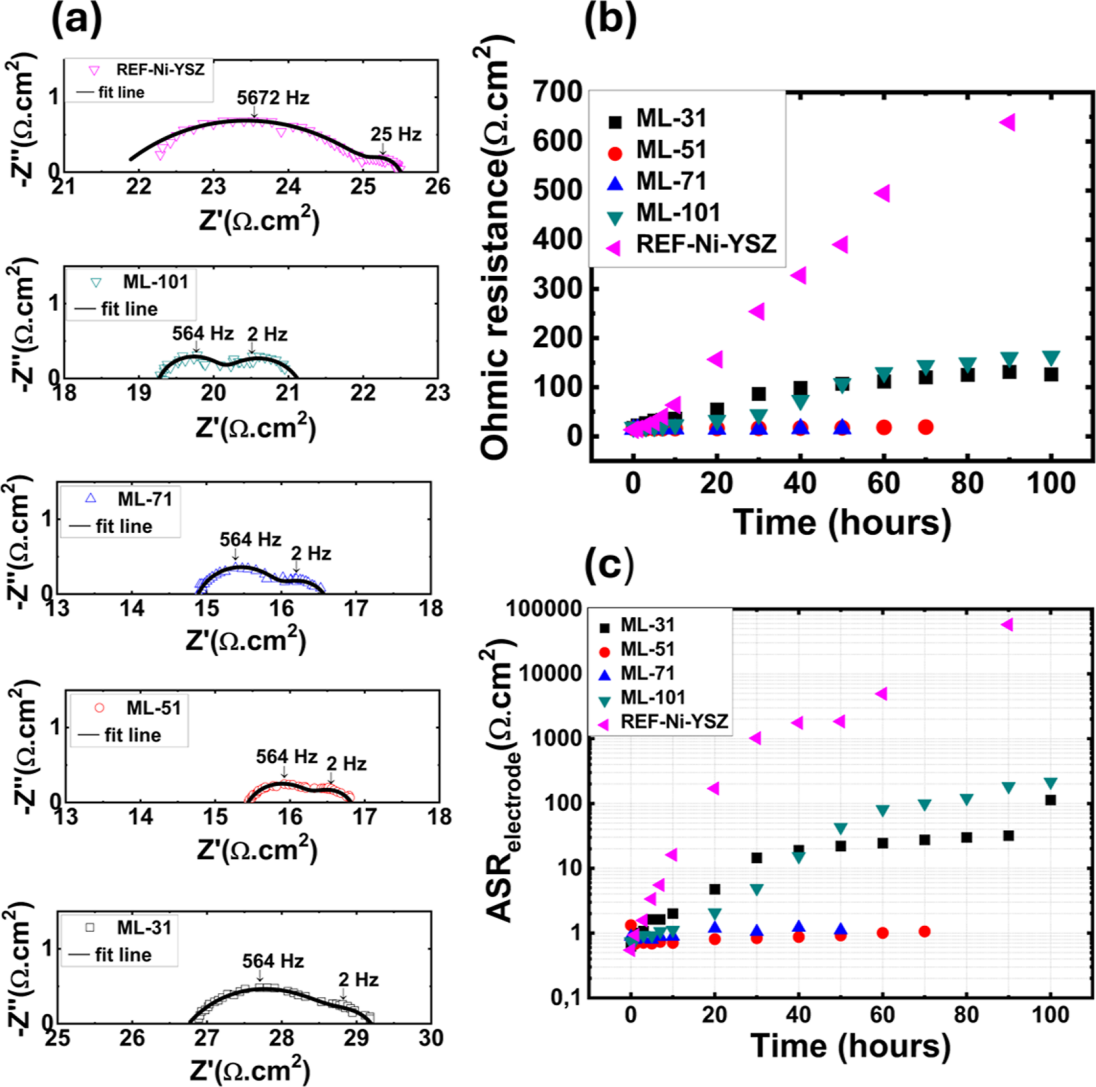
(a) Nyquist plots collected from REF-Ni–YSZ, ML-101, ML-71, ML-51, and ML-31 and changes in (b) ASR_electrode_ and (c) ASR_ohmic_ values, both extracted from EIS data, with time. The EIS measurements were conducted at an open-circuit voltage, at 650 °C, under 9.72% H_2_–87.51% Ar–2.77% H_2_O.

**Table 2:** Resistance, Frequency, and Capacitance Values Associated with the EIS Responses of Samples Extracted from Equivalent Circuit Fittings^a^

sample	ASR_ohmic_ (Ω·cm^2^)	ASR1 (Ω·cm^2^)	ASC1 (F/cm^2^)	f1 (Hz)	ASR2 (Ω·cm^2^)	ASC2 (F/cm^2^)	f2 (Hz)	ASR_total_ (Ω·cm^2^)	ASR_electrode_ (Ω·cm^2^)
REF-Ni–YSZ-3h	22.2	2.626	1.36 × 10^–8^	4.45 × 10^6^	0.642	3.28 × 10^–3^	7.56 × 10^1^	3.267	1.634
ML-101-3h	19.2	1.050	3.24 × 10^–6^	4.67 × 10^4^	0.833	3.615 × 10^–2^	5.285 × 10°^0^	1.883	0.942
ML-71-3h	14.9	1.050	1.36 × 10^–5^	1.11 × 10^4^	0.530	2.914 × 10^–2^	1.030 × 10^1^	1.581	0.790
ML-51-3h	16.8	0.851	1.18 × 10^–5^	1.59 × 10^4^	0.562	2.429 × 10^–2^	1.166 × 10^1^	1.413	0.707
ML-31-3h	25.7	1.853	3.02 × 10^–8^	2.84 × 10^6^	0.490	2.110 × 10^–2^	1.54 × 10^1^	2.342	1.171

a ASC and *f* values of the electrochemical processes were determined via ASC = (ASR × *Q*)^(1/*n*)^/ASR and *f* = 1/(2 × π × ASR × ASC), where *Q* and *n* are the fit parameters representing the constant-phase element and the deviation of the impedance arc shape from a perfect semi-circle, respectively.

After 3 h of exposure to 650 °C, ASR_electrode_ values of 1.634, 0.942, 0.790, 0.707, and 1.171 Ω·cm^2^ were obtained from REF-Ni–YSZ, ML-101, ML-71, ML-51, and ML-31, respectively (Figure 4a and Table 2). Upon 90 h exposure to dilute hydrogen at 650 °C, 700-fold increase in the ASR_ohmic_ and 10^5^-fold increase in the ASR_electrode_ values are observed in REF-Ni–YSZ (Figure 4b and c). Introduction of multiple CeO_2_ interlayers decreased the rate of performance loss (Figure 4b and c). Most stable performances were achieved in the case of ML-51 and ML-71, both reaching ASR_electrode_ values of 1.06 Ω·cm^2^, but the former in 60, latter in 70 h (Figure 4b and c). Faster performance degradation was observed when the number of CeO_2_ interlayers was further increased (Figure 4b and c).

The thickness of the applied CeO_2_ interlayers is another parameter that can influence the rate of Ni agglomeration and hence that of performance degradation. Considering that ML-51 exhibited performance stability superior to that of the other samples; to determine the effect of CeO_2_ interlayer thickness, this sample was taken as the baseline. Ni–YSZ electrodes with thicker CeO_2_ interlayers were fabricated by performing three deposition/drying cycles to form CeO_2_ interlayers every five Ni–YSZ deposition/drying cycles (ML-53). This way, the thickness of each CeO_2_ interlayer was increased while keeping the number of interlayers the same. Also considering that thicker CeO_2_ interlayers may interrupt electronic transport, electrodes with minor amounts of the Ni phase introduced into the thick CeO_2_ interlayers (i.e., 70:30 volume ratio of CeO_2_:Ni) were also fabricated (sample ML-53-Ni) by adding appropriate amounts of the Ni precursor into the CeO_2_ precursor (Figure 5).

**Figure 5:**
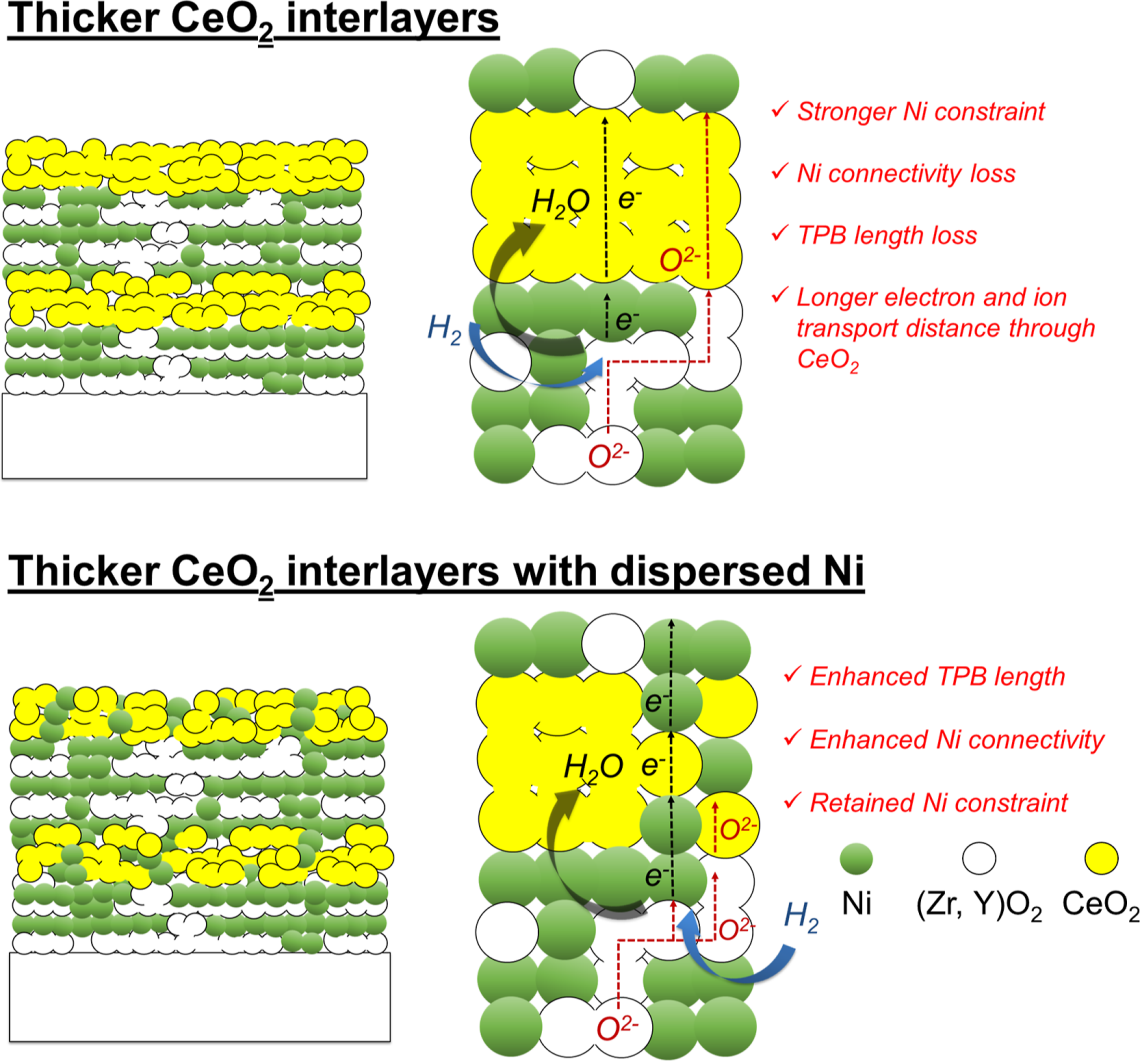
Schematic representation of the proposed hypothesis that performance degradation mechanisms in fuel electrodes will be prevented by multiple interlayers. While thicker CeO_2_ interlayers should provide a stronger mechanical constraint to Ni agglomeration, Ni particles interspersed into them should facilitate electronic conduction and thus minimize ohmic loss.

SEM analyses of nanoscale Ni–YSZ films with multiple, thick CeO_2_ interlayers with or without dispersed Ni phases (ML-53 and ML-53-Ni) revealed that cracks were present in both electrodes after 2 h of exposure to dilute hydrogen at 650 °C (Figure 6). Upon long-term exposure to the same conditions, cracks grew in number and width in both samples. A few micron-sized Ni particles appeared at the surface (Figure 6).

**Figure 6:**
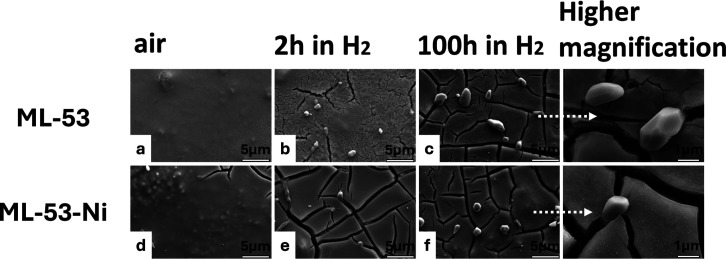
Top surface SEM images of ML-53 and ML-53-Ni in their as-calcined (a,d) and as-reduced states (b,e) and those collected after 100 h (c,f) of exposure to fuel gas (9.72% H_2_–87.51% Ar–2.77% H_2_O) at 650 °C.

STEM–EDS elemental maps were collected at the cross-section of the layered region of ML-53-Ni to analyze the morphological and compositional outcomes of the Ni-containing, thicker CeO_2_ interlayer application strategy (Figure 7). In its long-term annealed state, ML-53-Ni had CeO_2_ interlayers with ca. 80 nm thicknesses, with each layer consisting of three separate sublayers (Figure 7a). EDS maps collected at the higher magnification at the CeO_2_ interlayers also show the Ni particles interspersed within the CeO_2_ phase (Figure 7b). Furthermore, throughout the electrode, the Ni phase evidently was distributed in the form of thin layers up to ca. 27 nm thickness (Figure 7a).

**Figure 7:**
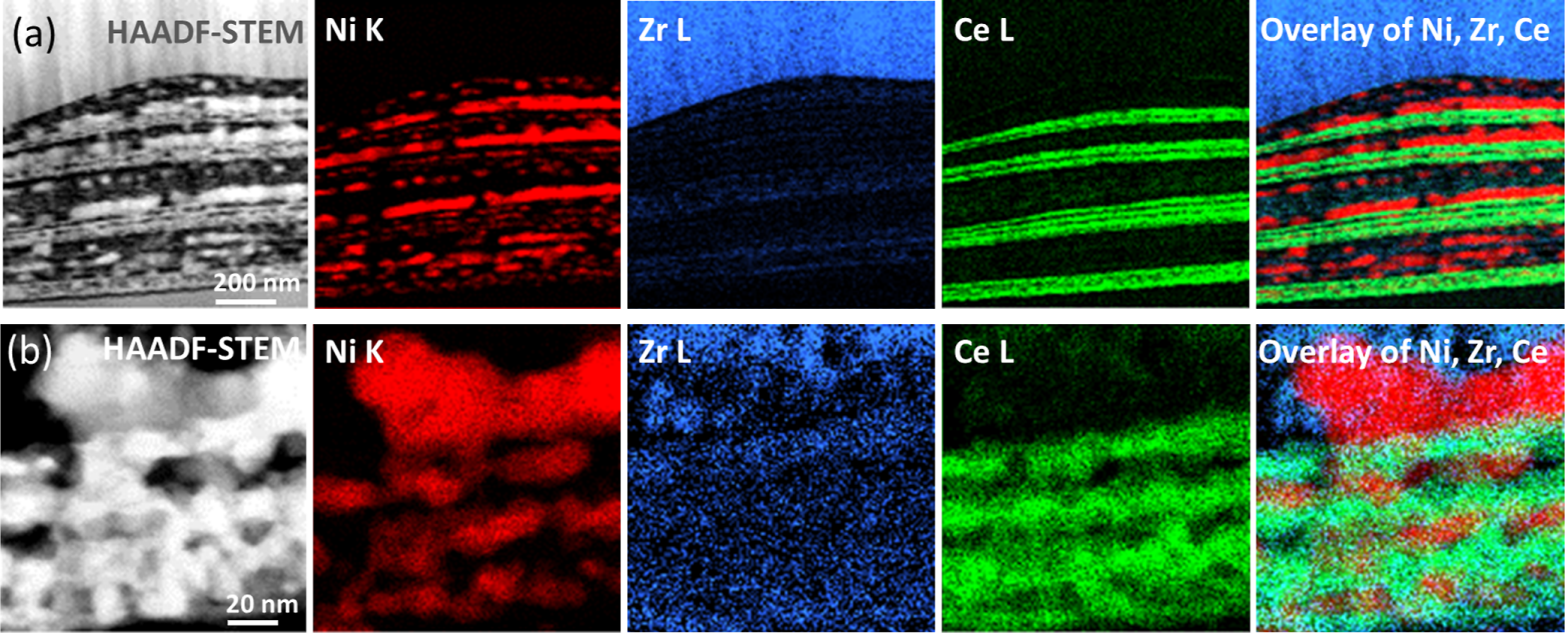
STEM–EDS maps and the corresponding HAADF-STEM images acquired from the cross-section of ML-53-Ni, exposed to 9.72% H_2_–87.51% Ar–2.77% H_2_O at 650 °C for 100 h. Top (a) and bottom (b) rows show low- and high-magnification images, respectively.

EIS measurements showed that the electrochemical performances of ML-53 and ML-53-Ni were vastly different (Figure 8). Fitting of the EIS data collected from ML-53-Ni was performed using the same equivalent circuit as the other samples; i.e., two resistor-constant phase element couples were connected in series to a single resistor. Meanwhile, an additional resistor-constant phase element couple was required for ML-53 (Table S1). Nyquist plots in Figure 8a after 3 h of exposure to measurement conditions revealed ML-53 ASR_ohmic_ and ASR_electrode_ values of 57.8 and 5.53 Ω·cm^2^, respectively (Figure 8a). Upon long-term exposure to 650 °C, under a dilute hydrogen flow, the ASR_electrode_ value of ML-53 increased significantly and then stabilized at ca. 26 Ω·cm^2^ (Figure 8b). ASR_ohmic_ also increased dramatically, up to ca. 140 Ω·cm^2^ (Figure 8c). On the other hand, ML-53-Ni exhibited much lower ASR_electrode_ values of 13.3 and 0.717 Ω·cm^2^, both of which remained somewhat stable during long-term testing (Figure 8b).

**Figure 8:**
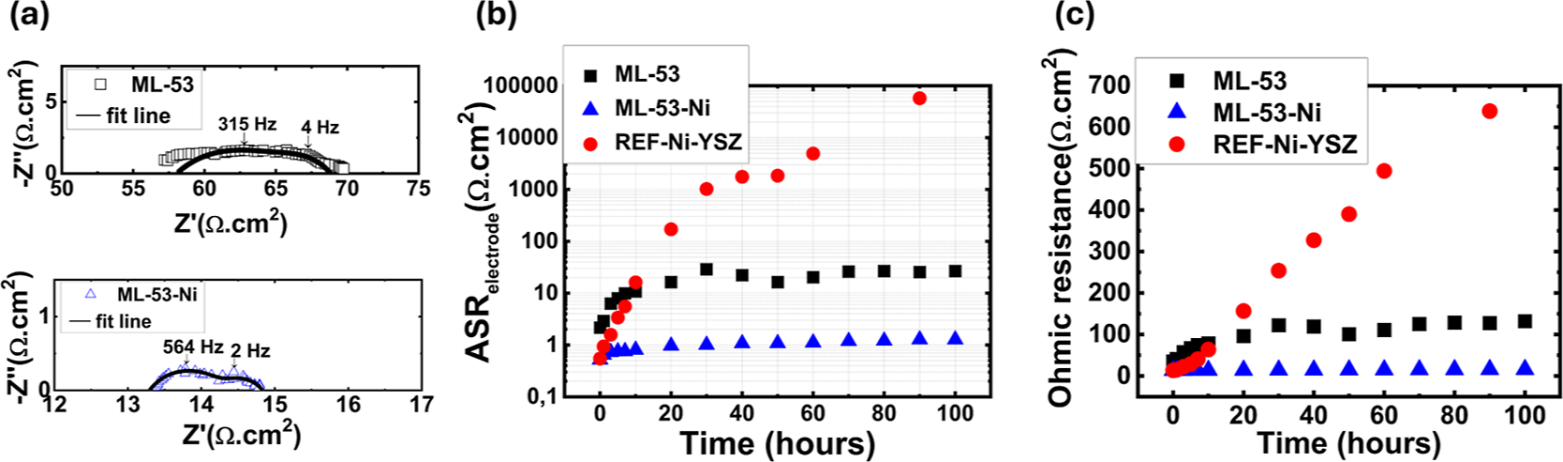
(a) Nyquist plots collected from ML-53 and ML-53-Ni after 3 h of exposure to operating conditions and changes in (b) ASR_electrode_ and (c) ASR_ohmic_ values, both extracted from EIS data, with time. The EIS measurements were conducted at an open-circuit voltage, at 650 °C, under 9.72% H_2_–87.51% Ar–2.77% H_2_O gas mixture.

## 4 Discussion

The presence of surface cracks in the as-reduced state of REF-Ni–YSZ (Figure 2f) suggests that Ni agglomeration had already begun as the electrode was heated up to 650 °C and soaked at that temperature for 2 h under a dilute hydrogen flow. The strong inverse correlation between the number of applied CeO_2_ interlayers and the number and size of surface cracks in the as-reduced state (Figure 2f, g, h, and i) suggests that the interlayers prevented Ni agglomeration. The fact that the large surface particles observed after long-term annealing were colocated with the cracks formed in the as-reduced state (Figure 2k, l, and m) indicates that the cracks acted as facile pathways for Ni to migrate out to the surface and reduce its surface area without being mechanically constrained. Prevention of Ni-agglomeration-induced surface crack formation evidently also inhibited Ni migration and formation of large-surface Ni particles.

Large differences in the thermal expansion coefficients (TECs) of the components of a multiphase system can cause cracking, especially in the case of a fabrication route that involves multiple deposition/drying (heat up/cool down) cycles. As the reviewer mentioned, in the present case, the addition of a third component (CeO_2_ interlayers) to a two-phase composite (NiO–YSZ) repetitive deposition/drying cycles, followed by precalcination at 800 °C, in air, could potentially cause crack formation, which, in turn, could act as facile routes for Ni agglomeration/migration during long-term testing.

Mori et al. measured the TEC values of YSZ, NiO, and NiO–YSZ as 11.2 × 10^–6^, 14.8 × 10^–6^, and 12.5 × 10–6 K^–1^, respectively, at 800 °C.^34^ At the same temperature, the TEC of undoped CeO_2_ was reported as 12.8 × 10–6 K^–1^.^35^ The fact that the TECs of the NiO–YSZ composite and CeO_2_ at the calcination temperature are very close to each other suggests that the TEC mismatch-induced crack formation is not expected for the nanoscale Ni–YSZ electrodes with multiple CeO_2_ interlayers. This is also supported by the absence of cracks in the as-fabricated states of the electrodes regardless of the number of CeO_2_ interlayers (Figure 2). The root cause of crack formation upon reduction is the stresses caused by fast Ni agglomeration and expansion of CeO_2_ caused by Ce^4+^/Ce^3+^ transition in the case of the maximum number of CeO_2_ interlayers, ML-31 (Figure 2).

Achievement of surfaces free of cracks and Ni agglomerates (even after long-term annealing) in the case of ML-51 (Figure 2i and n) is indicative of the effectiveness of the multiple oxide nanolayer approach. Meanwhile, the formation of cracks in the as-reduced state and the growth of large surface Ni agglomerates upon long-term annealing in the case of ML-31 (Figure 2j and o) indicate that there is more than one mechanism in play that may affect crack formation. One such mechanism is likely the expansion of CeO_2_ that occurs upon the reduction of Ce^4+^ to Ce^3+^ when exposed to dilute hydrogen,^36,37^ which evidently becomes dominant when the total CeO_2_ amount within the electrode coating exceeds a certain limit.

Electrode microstructures with horizontally aligned Ni and YSZ nanolayers stacked in an alternating fashion observed in TEM/EDS analyses (regardless of whether the samples had CeO_2_ interlayers or not, Figure 3a and b) were reported recently.^17^ It was argued that they originated from the differences in surface energies of NiO and YSZ and the absence of kinetic barriers when very thin depositions were made at each cycle.^17^ TEM/EDS showing thinner Ni layers in the presence of CeO_2_ interlayers after long-term annealing (Figure 3a and b) suggests that the Ni agglomeration and its prevention by CeO_2_ interlayers was not a phenomenon that was confined to the surface, but it was also observable within the electrode films.

The similar porosities of the microstructurally stable ML-51 and REF-Ni–YSZ (Figure S2) suggest that the Ni that migrated out to the surface was not in large enough amounts to significantly affect the overall porosity, at least in the analyzed area. However, the fact that the sizes of the pores were larger in the case of REF-Ni–YSZ corroborates well with the microstructural coarsening observed in this sample.

EIS measurements conducted on symmetrical half-cells and the equivalent circuit fittings performed on the received data showed two electrochemical processes, each associated with the area-specific resistance and capacitances of ASR1/ASC1 and ASR2/ASC2, along with an ohmic resistance (ASR_ohmic_). The fact that similarly fabricated Ni–YSZ nanoscale electrodes had their EIS data more appropriate for fitting to three semicircles in our earlier work,^17^ rather than two in this case, is likely due to the higher measurement temperature in the present case, which may have resulted in the dominance of one process at the expense of another.

Evidently, ASR_ohmic_ and ASR1 depended strongly on the extent of Ni agglomeration, i.e., TPB length and Ni interconnectivity. Considering this tendency along with the relatively low capacitances (Table 2), it can be suggested that ASR1 corresponds to electron transfer reaction at the TPB.^38–40^ The increasing ASC1 trend with decreasing Ni agglomeration tendency (Table 2) is also in agreement with the Ni/YSZ double layer capacitance which scales with the Ni/YSZ interfacial area.^39^ This correlation further supports the assignment of the high frequency process to the TPB charge transfer process.

The low-frequency process had relatively high capacitances and did not vary systematically from sample to sample. In the literature, low-frequency arcs of EIS data collected from a variety of Ni–YSZ electrodes were ascribed to either gas diffusion or gas conversion.^38,39^ Since gas diffusion should be temperature independent, we have conducted EIS measurements at different temperatures during the heating process, but each sample had different temperature dependencies of low-frequency ASR (data not shown). In other words, one sample exhibited a strong dependence on temperature, while another sample had a weak temperature dependence in their low-frequency ASR. The root cause of this discrepancy was likely the dynamic nature of the nanoscale electrode microstructures and the ongoing NiO reduction process. The NiO phase was probably still undergoing reduction, and microstructural coarsening was also still taking place during EIS data collection at 550, 600, and 650 °C, especially in the samples that were prone to Ni agglomeration. Hence, unequivocal determination of the nature of the low-frequency process was not possible at this point.

Overall, changes in the ASR_electrode_ were dictated by the changes in ASR1 and thus TPB loss due to Ni agglomeration. After 3 h of exposure to 650 °C, the lowest ASR_electrode_ value was obtained in ML-51, 0.707 Ω·cm^2^, the electrode that exhibited the lowest degree of Ni agglomeration. ASR_electrode_ values varying between 0.65 and 0.1 Ω·cm^2^ were reported in the literature for Ni–YSZ electrodes fabricated by cosintering of NiO and YSZ powders or Ni infiltration into porous YSZ scaffolds.^9,41^ One of the best Ni–YSZ performances was reported by Park et al. for cosintered Ni–YSZ electrodes in contact with an Fe_2_O_3_-doped YSZ electrolyte.^42^ They reported an ASR_electrode_ value of 0.75 Ω·cm^2^ at 600 °C, under 97% H_2_–3% H_2_O flow.^42^ Considering that this value was obtained under the flow of fuel gas with significantly higher hydrogen concentrations in comparison to the present case, it can be suggested that the nanoscale electrodes presented here exhibit performances in the range of the best performing cosintered electrodes reported in the literature.

Long-term stabilities of ASR_ohmic_ and ASR_electrode_ were strongly correlated to microstructural stability. For example, REF-Ni–YSZ exhibited an extremely fast increase in both ASR_ohmic_ and ASR_electrode_, which was accompanied by Ni agglomerate formation through the cracks at the surface (Figure 2f and k) and overall Ni agglomeration within the electrode layer (Figure 3a). In the case of this sample, Ni agglomeration resulted in the disconnection of the electronically conductive Ni network and the loss of effective TPB, which diminished both ASR_ohmic_ and ASR_electrode_.

In the case of nanoscale Ni–YSZ electrodes with thicker CeO_2_ interlayers (ML-53 and ML-53-Ni), surface cracks were present in both the as-reduced and long-term annealed states (Figure 6a and b). It was suggested here that the surface cracks present in the as-reduced state of ML-31 originated from the volume expansion of CeO_2_ upon its partial reduction. Considering that ML-53 and ML-53-Ni had even larger amounts of CeO_2_ than did ML-31, a stronger impact of CeO_2_ expansion upon partial reduction can be expected on crack formation. Formation of Ni agglomerates was observed in both ML-53 and ML-53-Ni samples but to a much lesser extent in comparison to the cases of REF-Ni–YSZ, ML-101, and ML-31 (Figure 6a and b), indicating that the suppression of Ni agglomeration was enhanced upon increasing the thickness of the CeO_2_ interlayers. Meanwhile, cracks formed as a result of CeO_2_ reduction provided some pathways through which Ni could migrate (Figure 6a and b).

EIS analyses revealed that ML-53 had ASR_ohmic_ that is much larger than what was observed in comparison to other samples (Figure 8a), suggesting that increasing the thickness of CeO_2_ interlayers indeed introduced additional resistance to electron transport. ASR_electrode_ was also affected by the same phenomenon; i.e., interruption of the electronic pathways reduced the effective TPB length. On the other hand, introduction of small amounts of the Ni phase into the thick CeO_2_ interlayers reduced the ASR_ohmic_ and ASR_electrode_ values (Figure 8b), down to levels comparable to what was observed in ML-51. Enhanced performance stability of ML-53 in comparison to that of REF-Ni–YSZ suggests that thicker CeO_2_ interlayers provided some degree of mechanical constraint to Ni agglomeration (Figure 8c). The fact that even more stable performances were achieved from ML-53-Ni at significantly lower ASR_ohmic_ and ASR_electrode_ values suggest that in the case of ML-53, the poor interconnectivity of the Ni phase became even worse, as a result of thick CeO_2_ interlayers in the case of ML-53. As can also be seen very clearly in the TEM–EDS analyses (Figure 7), the addition of small amounts of Ni into the thick CeO_2_ interlayers was very effective at maintaining Ni interconnectivity throughout the electrode, even after 100 h of testing.

## 5 Summary

In solid oxide cells, the particle size of the electrocatalyst—ionic conductor composite electrodes—must be reduced down to tens of nanometers to ensure long TPBs and, hence, acceptable performance even at the intermediate temperature range. While Ni-based nanocomposite electrodes fabricated by various means have been reported to exhibit a high electrochemical performance, they underwent fast Ni agglomeration and, consequently, performance loss.

In the present study, the introduction of multiple CeO_2_ interlayers into the nanocomposite Ni–YSZ electrodes was proposed as a means to mechanically constrain Ni agglomeration and thus retain nanoscale morphology and high electrochemical performance. CeO_2_ was selected especially due to its mixed ionic–electronic conducting nature. Number of CeO_2_ interlayers and their thicknesses were varied to determine how they affect performance and microstructure stability. SEM analyses revealed that reference Ni–YSZ electrodes with no CeO_2_ interlayers (REF-Ni–YSZ) underwent severe cracking upon reduction of NiO to Ni, which provided fast pathways for Ni to migrate to the outer free surface and form large agglomerates. Ni agglomeration within the electrode layer was also verified with TEM–EDS. As a result, at 650 °C, extremely fast electrochemical performance loss (i.e., 700-fold increase in the ASR_ohmic_ and 10^5^-fold increase in the ASR_electrode_) was verified by EIS.

Addition of the optimum number of CeO_2_ interlayers prevented crack formation at the electrode surface and inhibited Ni agglomeration. Relatively stable electrochemical performances (i.e., ca. 50% increase in 60 h) while maintaining the initial electrochemical performances were achieved at 650 °C.

Increasing the thickness of the CeO_2_ interlayer caused a disconnection of the electronically conductive Ni pathways while inhibiting Ni particle growth. This corresponded to a significant increase in the ASR_ohmic_ and ASR_electrode_ values initially. Performance loss took place as the Ni disconnection process continued. Dispersion of small amounts of Ni into the thick CeO_2_ interlayers served to maintain Ni interconnection throughout the electrode while inhibiting Ni agglomeration. Similar electrochemical performance and long-term stability results to those obtained from electrodes with optimized thin CeO_2_ interlayers were achieved.

Overall, it was shown that the application of mixed ionic–electronic conductor oxide interlayers into nanoscale Ni–YSZ electrodes served well to inhibit Ni agglomeration and enhance their long-term performance stability. The outcomes of this study can be used to construct intermediate-temperature solid oxide cells with a high performance and long-term stability.

## Supplementary Material



## Notes

Number of CeO_2_ interlayer and Ni–YSZ layer depositions for each sample, TEM and corresponding ImageJ-processed images obtained after 100 h testing of the REF-Ni–YSZ and ML-51 electrodes, and equivalent circuit fit parameters obtained from the EIS responses of ML-53 and ML-53-Ni samples (PDF)
