# Supplementary material for: Introduction of Multiple CeO2 Interlayers to Avoid Ni Agglomeration in Nanoscale Ni–YSZ Solid Oxide Cell Hydrogen Electrodes
Source: ACS Appl Mater Interfaces. 2025 Jul 16;17(30):43059–69. doi: 10.1021/acsami.5c08989 (PMC12314861; doi:10.1021/acsami.5c08989)
Supplement: Supplementary file 1 [file am5c08989_si_001.pdf]

**Supporting Information**

# Introduction of Multiple $CeO_2$ Interlayers to Avoid Ni Agglomeration in Nanoscale Ni-YSZ Solid Oxide Cell Hydrogen Electrodes

Mustafa Ünsal Ünver[1], Sorour Semsari Parapari[2], Saso Sturm[2,3,4], Aligul Buyukaksoy[1*]

1 Gebze Technical University, Department of Materials Science and Engineering 41400, Kocaeli Turkey

2 Jozef Stefan Institute, Department for Nanostructured Materials, 1000 Ljubljana, Slovenia

3 Jozef Stefan International Postgraduate School, 1000 Ljubljana, Slovenia

4 Department of Geology, Faculty of Natural Sciences and Engineering, University of Ljubljana, 1000 Ljubljana, Slovenia

*Corresponding author e-mail: aligul@gtu.edu.tr

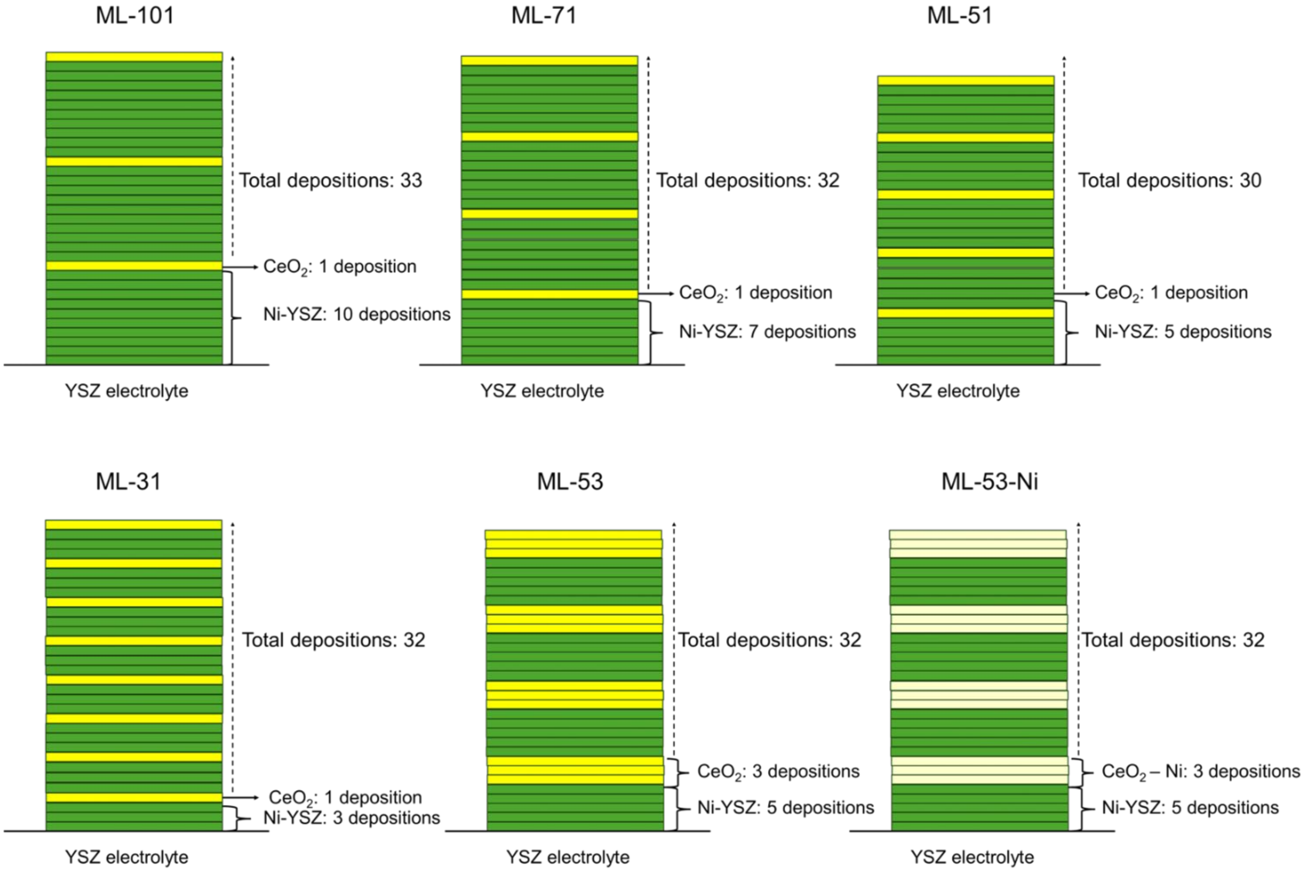


**Figure S1.** Schematic showing the number of $CeO_2$ interlayer and Ni-YSZ layer depositions for each sample.

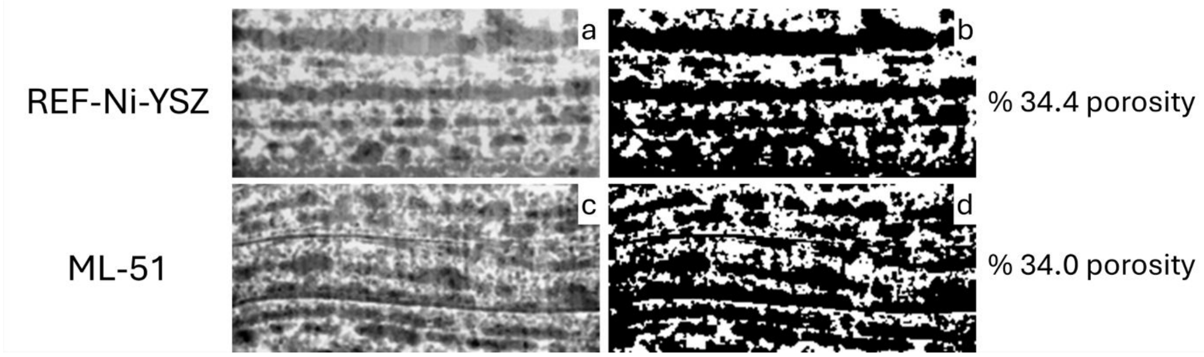


**Figure S2.** Bright-field STEM images of REF-Ni-YSZ (a) and ML-51 electrodes collected after 100 hours of testing and their corresponding binary images created by ImageJ.

**Table S1.** Resistance, frequency, and capacitance values associated with the EIS responses of ML-53 and ML-53-Ni extracted from equivalent circuit fittings.

| **Sample** | **ASRs** | **ASR1** | **ASC1** | **f1** | **ASR2** | **ASC2** | **f2** | **ASR3** | **ASC3** | **f3** | **ASRtotal** | **ASRelectrode** |
|---|---|---|---|---|---|---|---|---|---|---|---|---|
| ML-53-3h | 57.8 | 4.094 | 2.87E-08 | 1.36E+06 | 3.885 | 8.99E-06 | 4.56E+03 | 3.084 | 1.89E-03 | 2.736E+01 | 11.063 | 5.531 |
| ML-53-Ni-3h | 13.3 | 0.903 | 6.532E-06 | 2.698E+04 | 0.532 | 5.803E-02 | 5.160E+00 | | | | 1.435 | 0.717 |
